# Identifying subtle differences : a radiomics model assessment for gastric schwannomas and gastrointestinal stromal tumors across risk grades

**DOI:** 10.3389/fonc.2024.1467665

**Published:** 2024-12-18

**Authors:** Zimei Yang, Chongfei Ma, Jialiang Ren, Min Li, Xiaosheng Xv, Xin Fu, Li Yang

**Affiliations:** ^1^ Department of Computed Tomography and Magnetic Resonance, Fourth Hospital of Hebei Medical University, Shijiazhuang, Hebei, China; ^2^ Department of Pharmaceuticals Diagnostics, GE HealthCare, Beijing, China

**Keywords:** gastrointestinal stromal tumor, risk stratification, computed tomography imaging, gastric schwannoma, radiomics

## Abstract

**Objective:**

This study aims to develop and validate an enhanced computed tomography (CT)-based radiomics model to differentiate gastric schwannomas (GS) from gastrointestinal stromal tumors (GIST) across various risk categories.

**Methods:**

This retrospective analysis was conducted on 26 GS and 82 GIST cases, all confirmed by postoperative pathology. Data was divided into training and validation cohorts at a 7:3 ratio. We collected patient demographics, clinical presentations, and detailed CT imaging characteristics. Through univariable and multivariable logistic regression analyses, we identified independent predictors for discriminating between GS and GIST, facilitating the construction of a conventional model. Radiomic features were extracted and refined through manual 3D segmentation of venous phase thin-slice images to develop a radiomics model. Subsequently, we constructed a comprehensive combined model by integrating selected clinical and radiomics indicators. The diagnostic performances of all models in differentiating GS from GIST and stratifying GISTs according to malignancy risk were evaluated.

**Results:**

We identified several key independent variables distinguishing GS from GIST, including tumor location, cystic changes, degree of enhancement in arterial phase, and enhancement uniformity. The conventional model achieved AUCs of 0.939 and 0.869 in the training and validation cohort, respectively. Conversely, the radiomics model, predicated on eight pivotal radiomics features, demonstrated AUCs of 0.949 and 0.839. The combined model, incorporating tumor location, degree of enhancement in arterial phase, enhancement uniformity, and a radiomics model derived rad-score, significantly outperformed the traditional approach, achieving AUCs of 0.989 and 0.964 in the respective cohorts. The combined model showed superior diagnostic accuracy in distinguishing GS from GIST, as well as GS from high or low malignancy potential GISTs, as evidenced by IDI values of 0.2538, 0.2418, and 0.2749 (P<0.05 for all).

**Conclusion:**

The combined model based on CT imaging features and radiomics features presents a promising non-invasive approach for accurate preoperative differentiation between gastric schwannomas and gastrointestinal stromal tumors.

## 1 Introduction

Gastric schwannomas (GS), arising from Schwann cells in the submucosal nerve plexus of the stomach, are exceedingly rare tumors that typically present as benign lesions with favorable prognoses. Options for management include surgical resection or observational follow-up (1). In contrast, gastrointestinal stromal tumors (GIST), constitute the most prevalent mesenchymal neoplasms of the gastrointestinal tract. GISTs exhibit varying degrees of malignant potential, with higher risk classifications correlating with worse outcomes. While surgical resection remains the primary treatment approach, targeted therapy is indicated when complete (R0) resection is unfeasible, surgical risks are excessive, or extensive multi-organ resections would be required (2, 3).

The distinct therapeutic approaches and prognostic implications of GS versus GIST underscore the importance of accurate preoperative diagnosis. Current approaches for differentiating GS from GIST include conventional imaging techniques (CT, MRI), endoscopic ultrasound (EUS), fine-needle aspiration (FNA) biopsy, and immunohistochemistry. However, each method has significant limitations. Both tumor types present as submucosal gastric masses with overlapping endoscopic and radiographic appearances features, making differentiation challenging. Conventional techniques demonstrate limited specificity due to overlapping features, with accuracy rates of 60-80% (1). While EUS provides detailed visualization, its accuracy reaches only 70-75% (4). Although immunohistochemistry offers definitive diagnosis, it requires invasive tissue sampling. Moreover, the low success rate of biopsy via endoscopy further challenges preoperative discrimination, frequently leading to misdiagnosis of GS as GIST (5). These approaches are further constrained by their invasiveness nature, operator dependency, and limited accessibility in some healthcare settings. Given these constraints, there is a compelling need for diagnostic tools that are non-invasive, accurate, and widely applicable. Our proposed radiomics-based approach aims to address these limitations by leveraging advanced image analysis techniques to extract and analyze subtle imaging features beyond human visual perception, potentially improving preoperative differentiation between GS and GIST.

Radiomics, which involves the high-throughput extraction of quantitative features from medical images, has demonstrated promising results in the diagnosis and differentiation of gastrointestinal tumors (6, 7). This study aims to construct and evaluate a conventional model based on clinical and enhanced CT features, a radiomics model derived from venous phase imaging characteristics, and a combined model combining both. We assess their diagnostic performance in distinguishing GS from GIST and in stratifying GISTs by risk level, with the ultimate goal of establishing a reliable, non-invasive method for preoperative differentiation.

## 2 Materials and methods

### 2.1 Demographic of patients

We conducted a retrospective analysis of patient records from August 2015 to November 2021, including cases of gastric schwannomas (GS) and gastrointestinal stromal tumors (GIST) confirmed by surgical pathology at our institution. Inclusion criteria were as follows (1): patients with no history of other tumors and who had not received antitumor treatments prior to surgery; (2) cases where the tumor was completely excised, with comprehensive postoperative pathological data available, and where the risk stratification for GIST patients was clearly determined; (3) patients who underwent preoperative abdominal enhanced CT scans with complete imaging data available. Cases were excluded if the tumor was inadequately visualized due to small size or insufficient gastric distension.

### 2.2 Image acquisition

CT examinations was performed using multiple imaging systems including: Revolution CT(GE HealthCare), Somatom Definition Flash CT(Siemens Healthcare) and Somatom Sensation Open CT(Siemens Healthcare). Patient preparation included a 4-6 hours fasting period. Ten minutes before the procedure, an intramuscular injection of 10 mg raceanisodamine was administered to reduce motion artifacts, followed by the oral intake of 800-1000 ml of warm water or two sachets of effervescent granules to distend the stomach. All scans were performed with patients in the supine position, covering the region from the diaphragmatic dome to the symphysis pubis, with breath-holding during image acquisition.

Following a standard unenhanced scan, a high-pressure injector was used to administer an intravenous bolus of nonionic contrast medium (Iohexol, Omnipaque, 350mgI/ml, GE HealthCare) at a dose of 1.5 ml/kg and a flow rate of 3.0 ml/s through the antecubital vein. The arterial and venous phase images were acquired at 35 and 70 seconds post-injection, respectively. The scanning parameters were standardized across platforms: tube voltage of 120 kV with automatic tube current modulation, a rotation time of 0.5 seconds per rotation, pitch values of 0.9 (Siemens Healthcare) and 0.992 (GE HealthCare), and a scan slice thickness of 5.0 mm with an interval of 5.0 mm. Images were reconstructed with a slice thickness of 1.0 mm (Siemens Healthcare) and 1.25 mm (GE HealthCare).

### 2.3 Development of the conventional model

We systematically collected patient demographics and clinical data, including age, gender, and the symptoms of gastrointestinal hemorrhage. CT imaging features were comprehensively evaluated for each case, encompassing tumor location, the longest diameter on axial images, morphology (shape and growth pattern), tumor borders, homogeneity of density, and the presence of ulcers, cystic changes, liquefactive necrosis, hemorrhage, and calcification. Enhancement patterns were quantitatively assessed through CT attenuation measurements in unenhanced, arterial, and venous phases, including the degree of enhancement in arterial and venous phases and enhancement uniformity.

Using the training cohort data, both univariable and multivariable logistic regression analyses were employed to identify independent predictors capable of differentiating GS from GIST. Variables were selected based on both statistical significance and clinical relevance to develop a robust conventional diagnostic model. This model was specifically designed to facilitate accurate preoperative differentiation between these tumor types in clinical practice.

### 2.4 Radiomics feature analysis and model development

Tumor segmentation was performed on venous phase thin-slice CT images using ITK-SNAP software (version 3.8.0). A radiologist with five years of experience in abdominal imaging diagnostics (ZMY), manually delineated the entire tumor volume (exclude gastric contents, perigastric fat, and adjacent vascular structures) on a slice-by-slice basis, while blinded to clinical information. The segmentation accuracy was subsequently verified by a senior radiologist with twenty years of experience in imaging diagnostics (LY) to ensure precision of the tumor delineation.

Image preprocessing was conducted using PHIgo-AK software (version 1.4.0, GE HealthCare), which incorporates PyRadiomics (version 3.0.0, available at https://github.com/AIM-Harvard/pyradiomics/tree/master). The preprocessing pipeline included image resampling to a uniform voxel size of 1×1×1 mm³ and discretization of image intensity into 25 gray levels. This standardized approach enable the extraction of comprehensive radiomic features, including three-dimensional morphological characteristics, first-order intensity histogram parameters, and textural features. Additional image filtering was applied to enhance feature extraction, allowing for the capture of higher-order features.

The analytic phase involved univariable and correlation analyses to identify features significantly associated with the differentiation between GS and GIST. We then employed the Least Absolute Shrinkage and Selection Operator (LASSO) regression followed by stepwise multivariable regression to select independent predictive features for the radiomics model.

A comprehensive combined model was developed by integrating the previously identified independent clinical predictors with the radiomics-derived score (rad-score) through multivariable analysis. Additionally, a nomogram was developed to visualize the probability of tumor classification, enhancing the practical utility of the model in clinical decision-making.

### 2.5 Statistical methods

Statistical analysis were performed using R software (version 4.1.0, available at https://www.rproject.org). The normality of continuous variables were assessed using the Shapiro-Wilks test. Normally distributed variables were compared using independent samples t-test, while non-normally distributed variables were analyzed using the Mann-Whitney U test. Categorical variables were examined via the Chi-square test or Fisher’s exact test, depending on the appropriateness for the data structure and distribution. Receiver Operating Characteristic (ROC) curve analysis was used to assess the diagnostic accuracy of each model in differentiating between GS and GIST. To compare the diagnostic effectiveness of the models, the Integrated Discrimination Improvement (IDI) index was employed, offering a quantitative assessment of improvement in model prediction. Furthermore, Decision Curve Analysis (DCA) was utilized to ascertain the clinical utility of the models by quantifying the net benefits at various threshold probabilities. Additionally, we validated each model’s effectiveness in distinguishing GS from GISTs across different risk stratifications to ensure comprehensive assessment of diagnostic capability and clinical applicability.

## 3 Result

### 3.1 Demographic of patients

Following inclusion and exclusion criteria, the final cohort comprised 26 cases of gastric schwannomas (GS), with 10 males and 16 females, averaging 61.12 ± 9.14 years in age; and 82 cases of gastrointestinal stromal tumors (GIST), including 34 males and 48 females, with an average age of 61.28 ± 9.35 years. All CT examinations demonstrated adequate gastric distention and optimal contrast enhancement with clear visualization of the tumors. All included cases exhibited diagnostic image quality without significant motion artifacts or beam-hardening artifacts that could affect tumor evaluation. To facilitate a comprehensive evaluation of the models developed, the patient cohort was stratified into a training cohort and a validation cohort at a ratio of 7:3. Specifically, the training cohort included 77 patients (19 GS cases and 58 GIST cases), while the validation cohort included 31 patients (7 GS cases and 24 GIST cases).

### 3.2 Development of the conventional model

In the training cohort, statistically significant differences were observed between GS and GIST in terms of tumor location, the longest diameter on axial images, homogeneity of density, cystic changes, liquefactive necrosis, unenhanced CT values, venous phase CT values, and enhancement uniformity (P < 0.05), while no significant differences were noted for other characteristics (P > 0.05), as detailed in **Table 1**. Based on clinical experience and previous literature (1, 8–10), degree of enhancement in arterial phase was also identified as a potential discriminative feature and included in the analysis. Multivariable analysis revealed four independent predictors for GS-GIST differentiation. The outcomes highlighted tumor location [OR (95%CI) = 83.010 (9.328-2249.559), P < 0.001], cystic changes [OR (95%CI) = 8.117 (0.872-181.855), P = 0.092], degree of enhancement in arterial phase [OR (95%CI) = 1.107 (1.005-1.249), P = 0.061], and enhancement uniformity [OR (95%CI) = 41.603 (6.407-494.992), P < 0.001] as independent predictors for differentiating GS from GIST. Based on these predictors, the conventional model was constructed, demonstrating robust diagnostic performance with AUCs of 0.939 in the training cohort and 0.869 in the validation cohort. The model also achieved accuracy, sensitivity, and specificity rates of 0.818, 0.776, 0.947, and 0.774, 0.750, 0.857 for training and validation cohorts, respectively.

**Table 1:** Comparison of clinical and CT features between GS and GIST patients.

Characteristic	GS(n=19)	GIST(n=58)	*p*-value
Gender			1.000
Female	11 (57.895%)	33 (56.897%)	
Male	8 (42.105%)	25 (43.103%)	
Age	60.000 [52.000;67.000]	63.000 [57.000;69.000]	0.315
Presence of gastrointestinal hemorrhage symptoms			0.747
No	16 (84.211%)	45 (77.586%)	
Yes	3 (15.789%)	13 (22.414%)	
Tumor Location			0.010
Fundus	1 (5.263%)	22 (37.931%)	
Body	13 (68.421%)	29 (50.000%)	
Antrum	5 (26.316%)	7 (12.069%)	
Longest diameter on axial images	3.300 [2.300;4.100]	4.700 [3.050;5.800]	0.037
Shape			0.372
Regular	16 (84.211%)	42 (72.414%)	
Irregular	3 (15.789%)	16 (27.586%)	
Growth pattern:			0.425
Intracavity	3 (15.789%)	18 (31.034%)	
Extraluminal	6 (31.579%)	16 (27.586%)	
Intracavity+ extraluminal	10 (52.632%)	24 (41.379%)	
Tumor border			1.000
Clear	12 (63.158%)	37 (63.793%)	
Blurred	7 (36.842%)	21 (36.207%)	
Homogeneity of density			0.001
Uniformity	14 (73.684%)	16 (27.586%)	
Nonuniformity	5 (26.316%)	42 (72.414%)	
Presence of ulcers			1.000
No	12 (63.158%)	38 (65.517%)	
Yes	7 (36.842%)	20 (34.483%)	
Presence of cystic changes			0.006
No	18 (94.737%)	33 (56.897%)	
Yes	1 (5.263%)	25 (43.103%)	
Presence of liquefactive necrosis			0.003
No	16 (84.211%)	24 (41.379%)	
Yes	3 (15.789%)	34 (58.621%)	
Presence of hemorrhage			1.000
No	19 (100.000%)	56 (96.552%)	
Yes	0 (0.000%)	2 (3.448%)	
Presence of calcification			1.000
No	16 (84.211%)	48 (82.759%)	
Yes	3 (15.789%)	10 (17.241%)	
Unenhanced CT values	36.000 [32.050;40.500]	32.150 [28.200;36.675]	0.026
Arterial phase CT values	52.800 [48.200;58.550]	50.350 [40.850;61.050]	0.603
Venous phase CT values	70.000 [60.000;79.800]	61.800 [53.225;69.875]	0.031
Degree of enhancement in arterial phase	16.500 [13.000;19.350]	17.650 [9.400;26.725]	0.531
Degree of enhancement in venous phase	31.700 [24.950;42.800]	27.650 [21.050;34.125]	0.091
Enhancement uniformity			<0.001
Uniformity	16 (84.211%)	16 (27.586%)	
Nonuniformity	3 (15.789%)	42 (72.414%)	

### 3.3 Development of the radiomics model

The radiomics features encompassed three primary categories of quantitative descriptors, including: (1) three-dimensional (3D) morphological features, capturing the geometric properties of the tumors; (2) first-order statistical features, reflecting the distribution of pixel intensities; and (3) higher-order textural features, which provided insights into the complexity of tumor texture through various matrices such as the Gray Level Cooccurrence Matrix (GLCM), Gray Level Run Length Matrix (GLRLM), Gray Level Size Zone Matrix (GLSZM), Gray Level Dependence Matrix (GLDM), and Neighbouring Gray Tone Difference Matrix (NGTDM).

**Figure 1:**
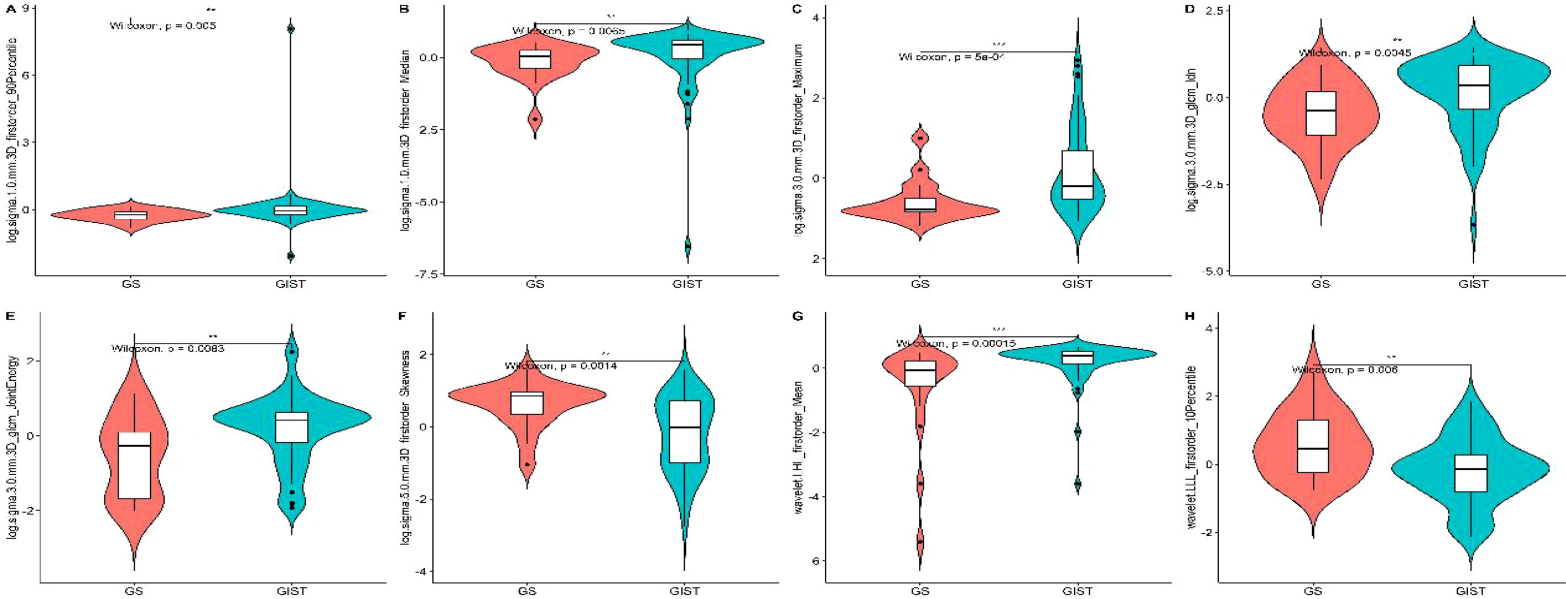
violin plots of radiomics features to demonstrate the distribution of values.

The initial feature extraction process yielded 1,595 features. After feature selection process, eight key radiomic features with optimal discriminative power were identified. These included six first-order statistical features and two derived from the GLCM, specifically: 1) log.1-firstorder-90Percentile; 2) log.1-firstorder-Median; 3) log.3-firstorder-Maximum; 4) log.3-glcm-Idn; 5) log.3-glcm-JointEnergy; 6) log.5-firstorder-Skewness; 7) wavelet.LHL-firstorder-Mean; 8) wavelet.LLL-firstorder-10Percentile. This selection underscored the critical role of intensity distribution and textural heterogeneity in distinguishing GS from GIST (**Figure 1**).

**Figure 2:**
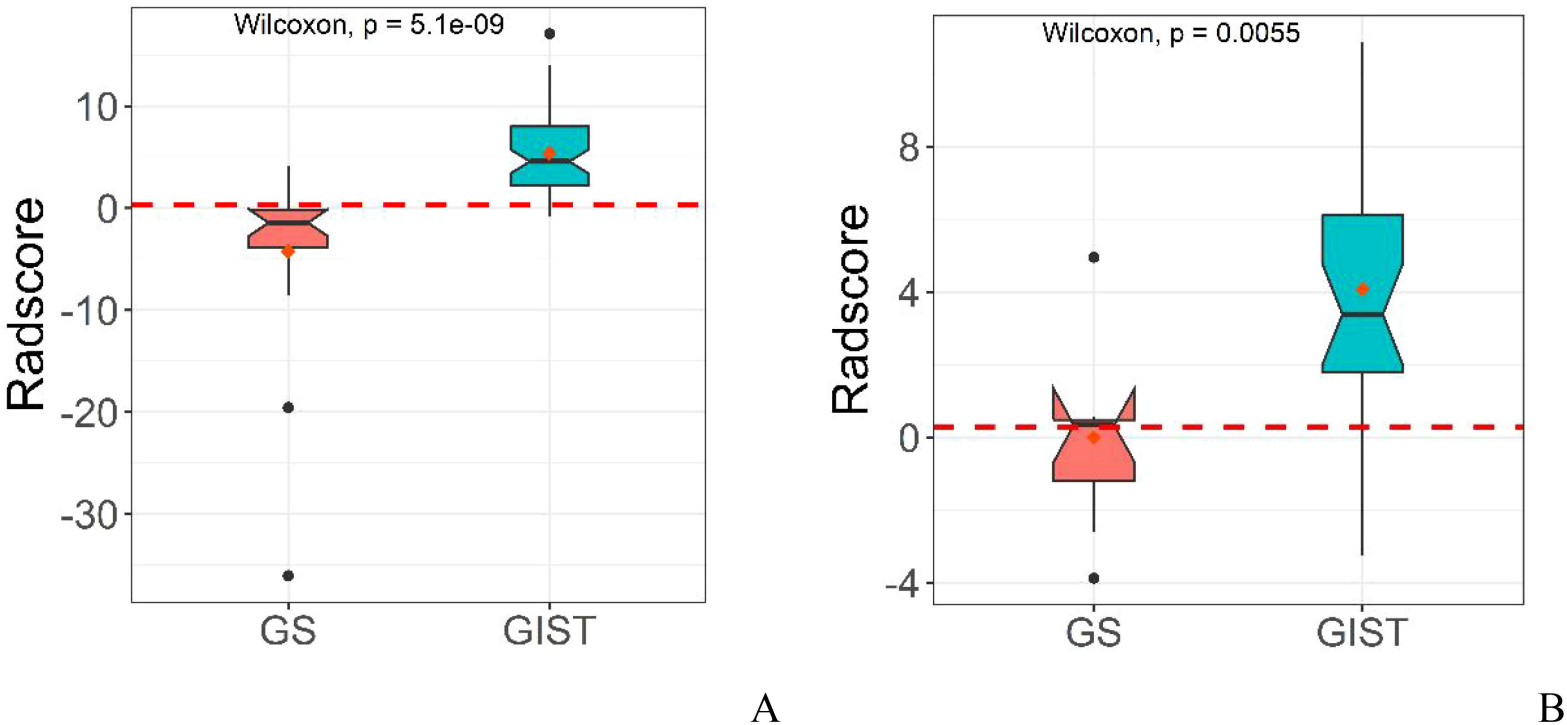
Distribution of rad-score in radiomics model in the training cohort **(A)** and validation cohort **(B)**.

Based on the selected radiomic features, we constructed a diagnostic model that generated a radiomics rad-score for each case (**Figure 2**). The radiomics model demonstrated exceptional diagnostic performance, with Area Under the Curve (AUC) values of 0.949 in the training cohort and 0.839 in the validation cohort. Moreover, the model achieved accuracy, sensitivity, and specificity rates of 0.922, 0.948, 0.842, and 0.774, 0.875, 0.429, respectively, highlighting its significant potential in clinical applications for the precise differentiation between GS and GIST.

### 3.4 Development of the combined model

Through multivariable analysis, we developed a combined model integrating the independent predictors from the conventional model and the rad-score derived from the radiomics model. The final model incorporated tumor location, degree of enhancement in arterial phase, enhancement uniformity, and the rad-score as key predictive factors. This comprehensive model demonstrated exceptional diagnostic performance, achieving AUC, accuracy, sensitivity, and specificity metrics of 0.989, 0.961, 0.966, 0.947 in the training cohort, and 0.964, 0.871, 0.917, 0.714 in the validation cohort, respectively.

**Figure 3:**
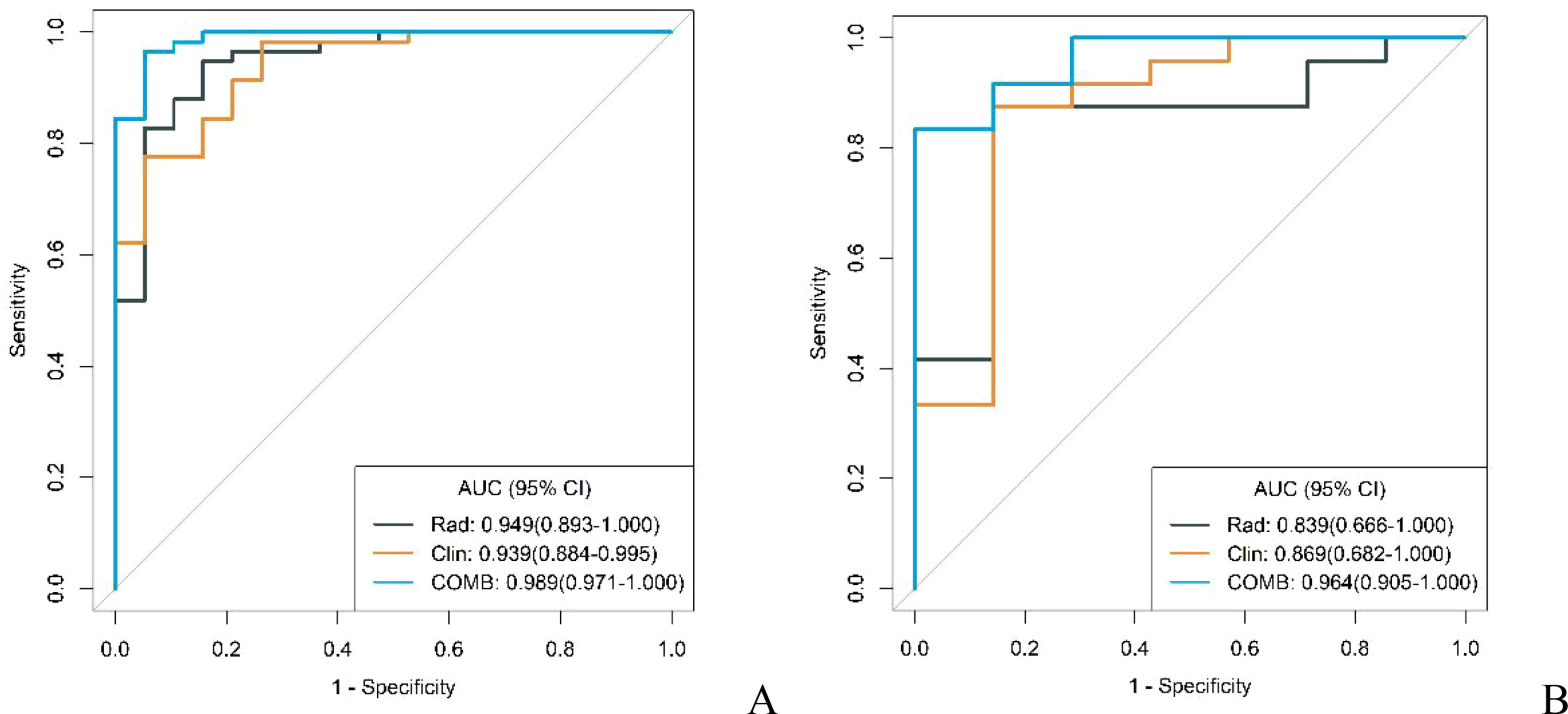
ROC curves of conventional model, radiomics model and combined model in the training cohort **(A)** and validation cohort **(B)**.

**Figure 4:**
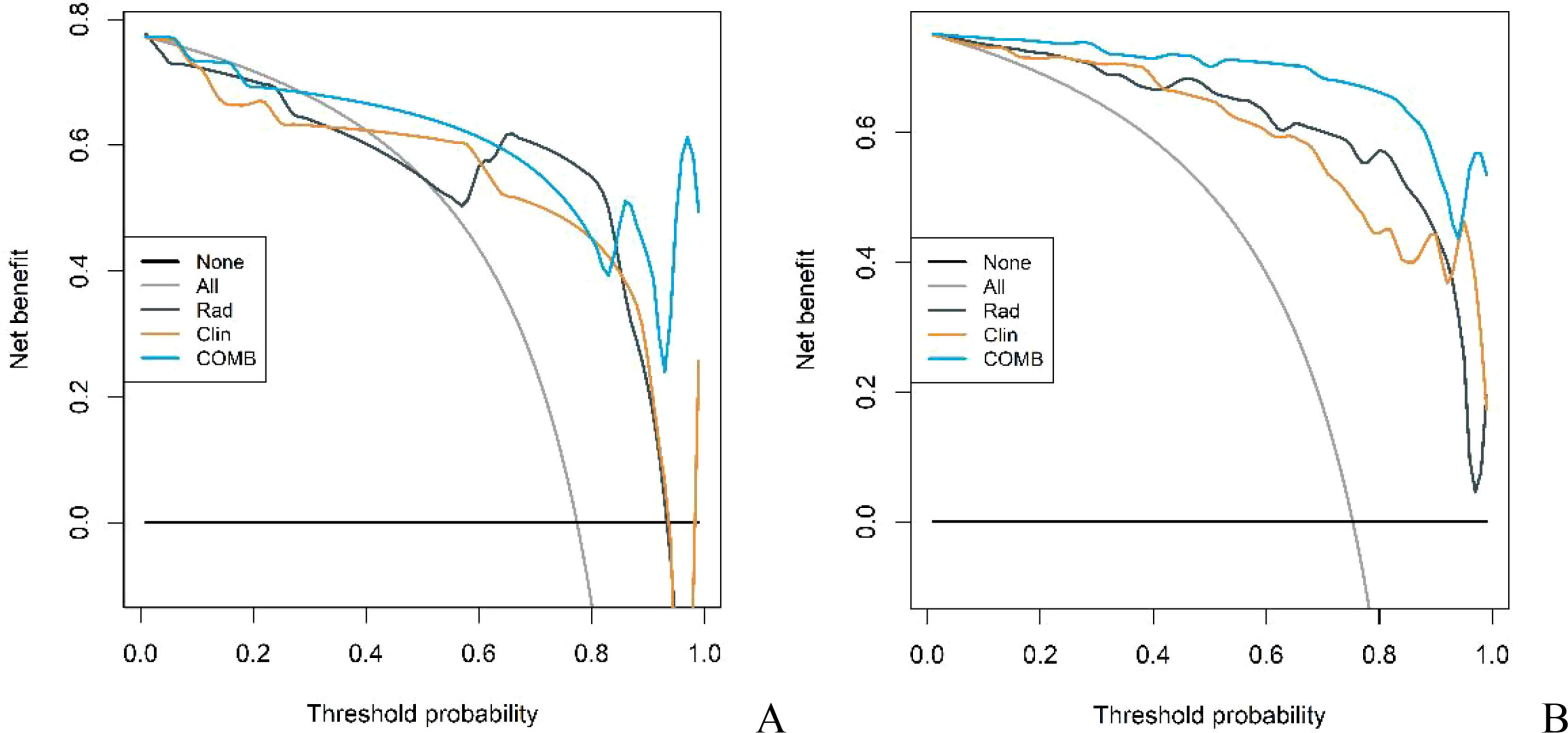
DCA curves of conventional model, radiomics model and combined model in the training cohort **(A)** and validation cohort **(B)**.

The diagnostic capabilities of the conventional model, the radiomics model, and the integrated (combined) model in distinguishing GS from GIST were visually represented through ROC curves (**Figure 3**). The combined model significantly outperformed the conventional model in diagnostic efficacy, as evidenced by an IDI of 0.2538 (P < 0.05). DCA revealed that while all three models provided clinical benefits above the baseline, the combined model consistently yielded the highest net clinical benefit across both training and validation cohorts, substantiating its superior clinical utility (**Figure 4**).

**Figure 5:**
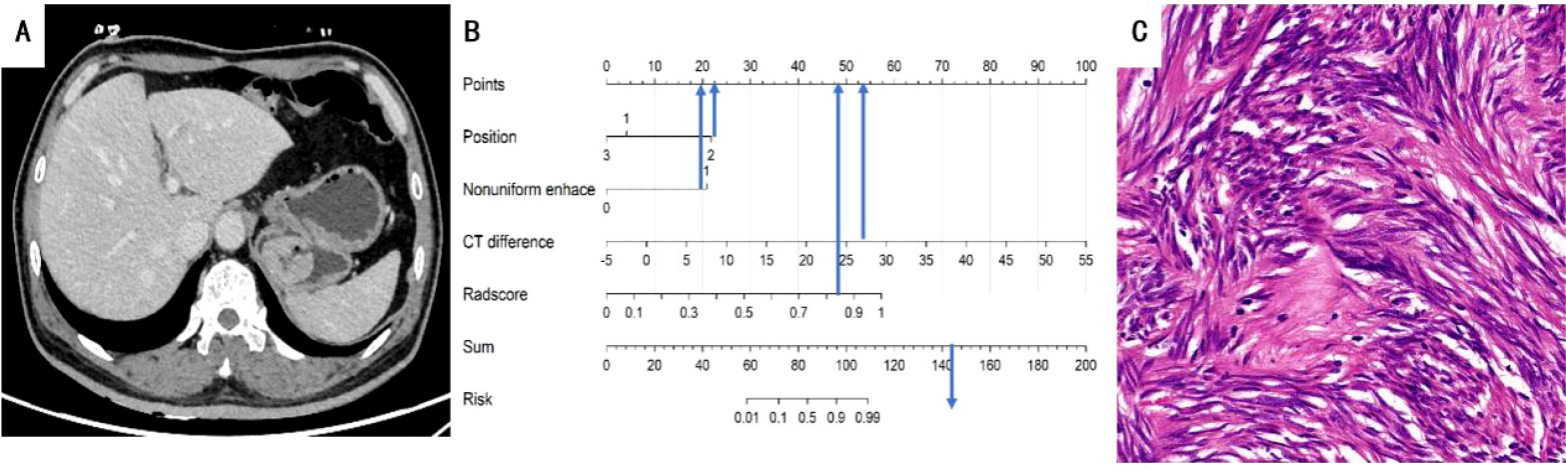
**(A)** A 63-year-old man with GIST. The axial CT image demonstrated a 3.8-cm-sized tumor in the fundus of the stomach. Axial CT image showed mucosal ulceration at the margin of the mass. **(B)** Total points of the tumor were 144, risk value was 1.6 and it was classified as GIST by the nomogram. **(C)** Pathological HE (×200) of the tumor.

**Figure 6:**
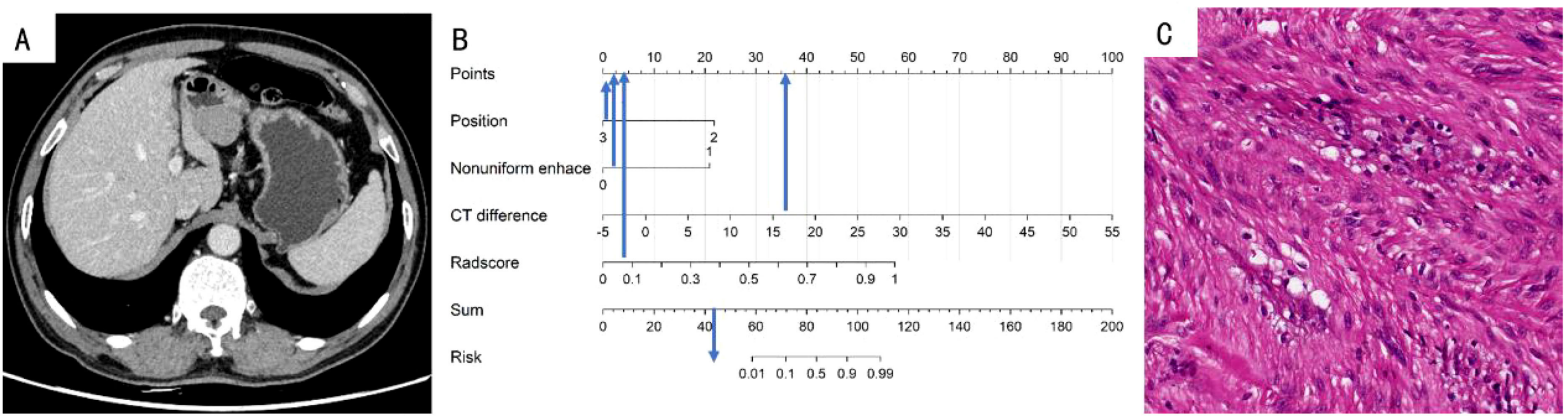
**(A)** A 70-year-old woman with GS. The axial CT image demonstrated a 3.4-cm-sized tumor in the antrum of the stomach. **(B)** Total points of the tumor was 44, risk value was far less than 0.1 and it was classified as GS by the nomogram. **(C)** Pathological HE (×200) of the tumor.

To facilitate clinical implementation, we transformed it into a nomogram, enabling individualized risk prediction and visual interpretation of results. The nomogram incorporated all significant predictive factors and provided a straightforward approach for calculating patient-specific probabilities. The nomogram’s critical threshold was established at 0.596: scores above this threshold suggest a diagnosis of GIST, while lower scores indicate GS. The detailed construction and application of the nomogram are illustrated in **Figures 5** and **6**, providing clinicians with a practical tool for quantitative risk assessment through the cumulative effect of individual predictive factors.

### 3.5 Diagnostic efficacy for identifying GS with GIST of different risk grades

Further analysis evaluated the models’ diagnostic performance in distinguishing gastric GS from GIST across different risk grades. Postoperative pathological assessment classified the 82 GIST cases were stratified into four risk categories: very low risk (7 cases), low risk (22 cases), intermediate risk (26 cases), and high risk (27 cases). For analytical purposes, very low-risk and low-risk cases were grouped into a low malignancy potential category (n=29), whereas intermediate-risk and high-risk cases were classified into a high malignancy potential category (n=53).

**Figure 7:**
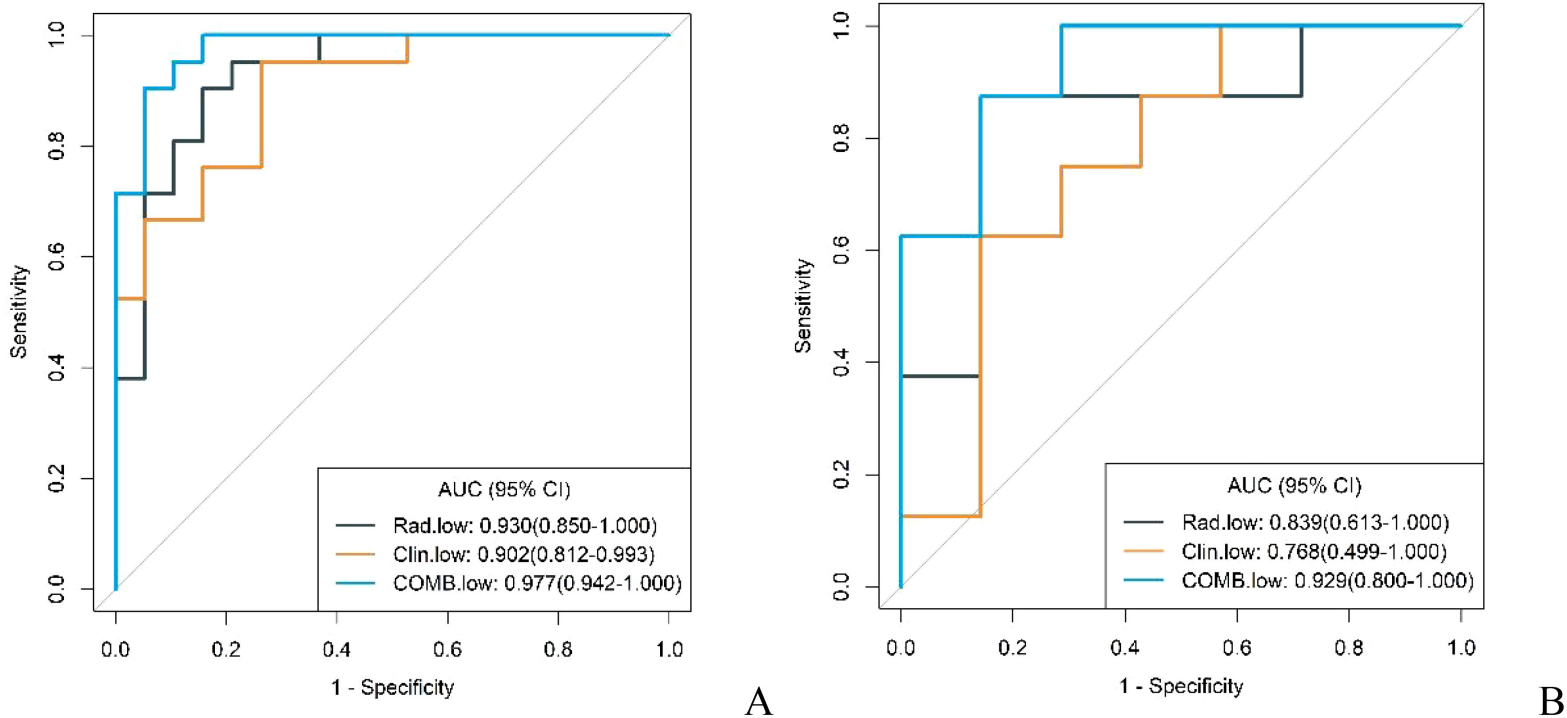
ROC curves for identification of GS with low malignant potential GIST by conventional model, radiomics model, and combined model in the training cohort **(A)** and validation cohort **(B)**.

**Figure 8:**
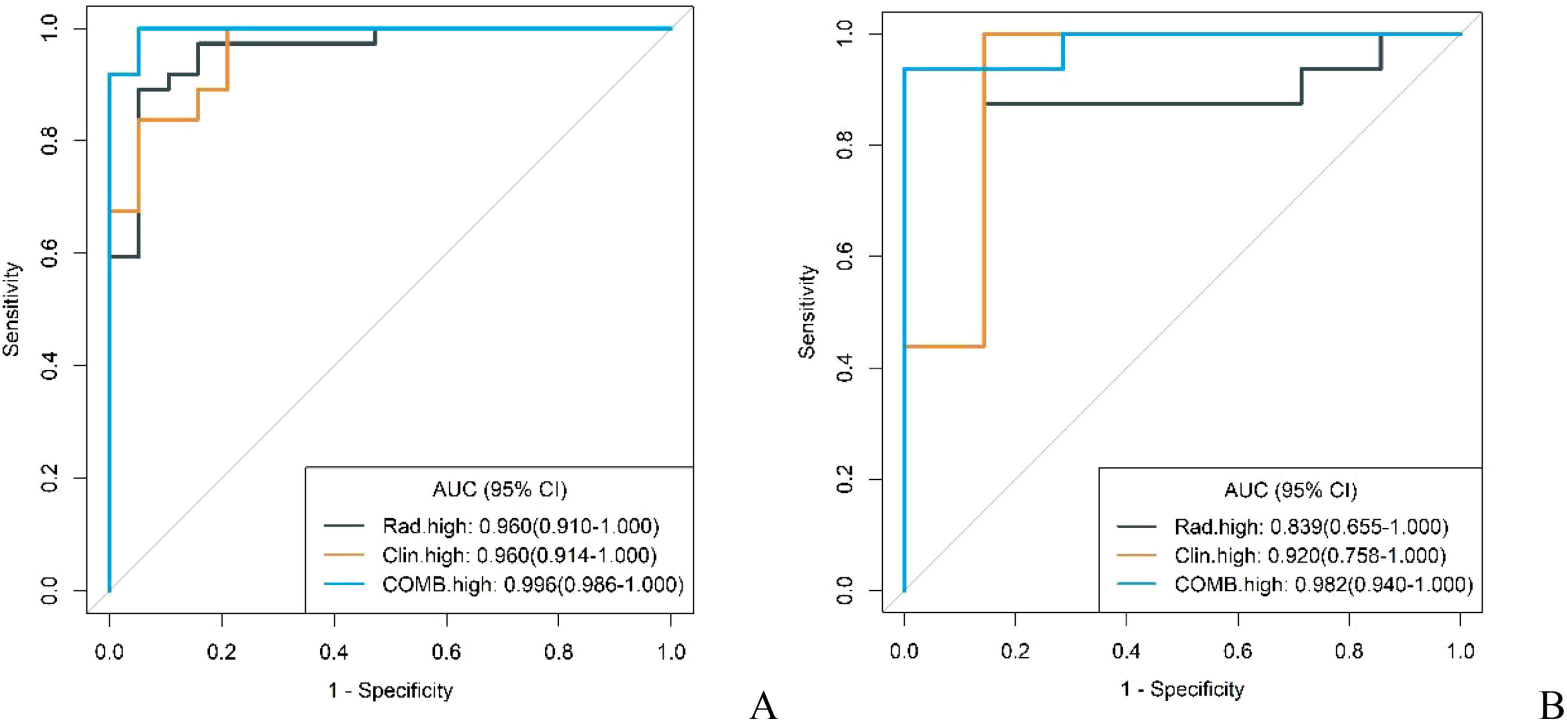
ROC curves for identification of GS with high malignant potential GIST by conventional model, radiomics model, and combined model in the training cohort **(A)** and validation cohort **(B)**.

In both the training and validation cohorts, the conventional model, radiomics model, and combined model demonstrated good diagnostic performance in differentiating GS from high malignancy potential GIST and low malignancy potential GIST. The AUC values in the training cohort were notably higher for distinguishing GS from high malignancy potential GIST than from low malignancy potential GIST (0.960 vs. 0.902; 0.960 vs. 0.930; 0.996 vs. 0.977, respectively). This pattern persisted in the validation cohort (0.920 vs. 0.768; 0.839 vs. 0.839; and 0.982 vs. 0.929, respectively) as shown in **Figures 7** and **8**. Notably, the combined model demonstrated superior diagnostic performance compared to the conventional model for both high and low malignancy potential GIST, with significant IDI values of 0.2418 and 0.2749, respectively (both P < 0.05).

## 4 Discussion

Accurate preoperative differentiation between GS and GIST is crucial for optimizing clinical management and treatment strategies. In this study, we developed and evaluated three distinct models based on contrast-enhanced CT imaging: a conventional model, a radiomics model, and a combined model integrating both approaches. Our findings reveal that the combined model, which incorporates both traditional CT imaging characteristics and radiomics features, achieves superior diagnostic performance compared to conventional methods, suggesting its potential as a non-invasive and precise method for accurately distinguishing between GS and GIST preoperatively.

Our study identified four traditional imaging features—tumor location, cystic changes, degree of enhancement in arterial phase, and enhancement uniformity—as independent predictors for differentiating GS from GIST. Among the GS cases in our cohort, a significant majority (20 out of 26, 76.92%) were located in the gastric body, with only one instance (1 out of 26, 3.85%) found in the gastric fundus. Conversely, of the GIST cases, 44 were identified in the gastric body (44 out of 82, 53.66%), and 27 in the gastric fundus (27 out of 82, 32.93%). These observations align with existing literature, which suggests that GS are predominantly located along the greater curvature of the gastric body, whereas GISTs are more commonly found in the gastric fundus or body (11).

GS typically display benign biological behavior, characterized by slow cellular proliferation and minimal degenerative changes, including cystic degeneration (12). In contrast, GIST demonstrates potential malignancy behavior with active cell proliferation. Even small-sized GISTs can develop hypoperfusion, leading to regressive changes within the tumor. Our study findings reflect these biological differences: cystic changes were observed in only 7.69% (2/26) of GS cases compared to 41.46% (34/82) of GIST cases. Furthermore, the regressive changes in GIST can promote arteriovenous shunt formation, heterogeneous arterial phase enhancement patterns due to irregular vascular density distribution and marked tumor heterogeneity (1, 13, 14). While PET/CT demonstrates excellent capability in differentiating benign from malignant tumors and detecting metastases (15, 16). However, due to the high cost and long time consuming of this examination method, it is still limited in the rapid and accurate identification of GS and GIST.

The radiomics model, based on high-throughput quantitative feature extraction, overcomes the inherent subjectivity of traditional diagnostic approaches (17, 18). This model provides objective quantification of tumor heterogeneity with unprecedented precision, enabling the capture of subtle imaging characteristics that may elude visual assessment. Through feature selection, eight radiomics features were identified as critical for the model construction, including six first-order intensity histogram features and two gray level co-occurrence matrix features. These features comprehensively characterize tumor heterogeneity by quantifying both the distribution of pixel intensities and their spatial interrelationships within the tumor volume.

Our analysis stratified GIST cases into high and low malignancy potential subgroups to evaluate the diagnostic performance of conventional, radiomics, and combined models across different risk grades. The combined model demonstrated superior diagnostic efficacy in differentiating GS from both high and low malignancy potential GIST, significantly outperforming the conventional model. Notably, the model achieved higher diagnostic accuracy in identifying high malignancy potential GIST, likely due to their distinct biological characteristics. These tumors typically exhibit aggressive growth patterns with more pronounced cystic changes, liquefactive necrosis, and increased heterogeneity (19, 20), creating more discernible imaging features compared to GS. In contrast, low malignancy potential GIST posed greater diagnostic challenges due to their less aggressive growth patterns and reduced heterogeneity, which more closely resemble the imaging characteristics of GS.

Several limitations of this study should be acknowledged. First, the retrospective, single-center of this study introduces potential selection bias and may limit result generalizability. Second, the inherent rarity of gastric schwannomas resulted in a relatively smaller sample size, which could affect the statistical power of our findings. We addressed this limitation by carefully selecting radiomics features and implementing subgroup validation to minimize model overfitting and enhance analytical robustness. Third, the utilization of different CT scanners introduced imaging heterogeneity. Although we employed standardized preprocessing protocols to mitigate this variation, its potential impact on results cannot be completely eliminated. Future research should focus on prospective, multicenter studies with larger cohorts to validate our findings. Such investigations would not only confirm the combined model’s efficacy across diverse clinical settings but also provide deeper insights into its role in treatment strategy optimization for both GS and GIST patients.

## 5 Conclusion

Our study presents a novel combined model integrating conventional CT imaging features with radiomics analysis for enhanced preoperative differentiation between gastric schwannomas and gastrointestinal stromal tumors. This comprehensive approach demonstrates superior diagnostic performance compared to traditional methods, offering a promising tool for more accurate preoperative diagnosis and optimized treatment planning.

## Data Availability

The original contributions presented in the study are included in the article/supplementary material. Further inquiries can be directed to the corresponding author.

## References

[1] Li R Gan H Ni S. Differentiation of gastric schwannoma from gastric gastrointestinal stromal tumor with dual-phase contrast enhanced computed tomography. J Comput Assist Tomogr. (2019) 43:741–6. doi: 10.1097/RCT.0000000000000902 31356524

[2] Miettinen M Majidi M Lasota J. Pathology and diagnostic criteria of gastrointestinal stromal tumors (GISTs): a review. Eur J Cancer. (2002) 38:S39–51. doi: 10.1016/S0959-8049(02)80602-5 12528772

[3] Nishida T Hirota S. Biological and clinical review of stromal tumors in the gastrointestinal tract. Histol histopathology. (2000) 15:1293–301. 10.14670/HH-15.1293 11005253

[4] Wanwan C Guoping C. Endoscopic ultrasound-guided fine-needle aspiration biopsy of gastric schwannoma: Cytomorphologic features and diagnostic pitfalls. Diagn cytopathology. (2019) 47:1218–22. 10.1002/dc.24289 31343112

[5] Guthrie G Mullen R Moses A. Gastric Schwannoma or GIST: accuracy of preoperative diagnosis? Scottish Med J. (2011) 56:1–3. 10.1258/smj.2011.011175 22089050

[6] Roberto C Ludovico LG Massimo M. New advances in radiomics of gastrointestinal stromal tumors. World J Gastroenterol. 26:4729–38. 10.3748/wjg.v26.i32.4729 PMC7459199 32921953

[7] Ba-Ssalamah A Muin D Schernthaner R. Texture-based classification of different gastric tumors at contrast-enhanced CT. Eur J Radiol. (2013) 82:e537–43. doi: 10.1016/j.ejrad.2013.06.024 23910996

[8] Wook JC Dongil C Kyoung-Mee K. Small submucosal tumors of the stomach: differentiation of gastric schwannoma from gastrointestinal stromal tumor with CT. Korean J Radiol. (2012) 13:425–33. doi: 10.3348/kjr.2012.13.4.425 PMC3384824 22778564

[9] Shah AS Rathi MP Somani SV. Gastric schwannoma: A benign tumor often misdiagnosed as gastrointestinal stromal tumor. Clinics Pract. (2015) 5:775. doi: 10.4081/cp.2015.775 PMC4653750 26664714

[10] Jianli L Yanjun C Junlin Z. Spectral computed tomography imaging of gastric schwannoma and gastric stromal tumor. J Comput assisted tomography. (2017) 41:417–21. doi: 10.1097/RCT.0000000000000548 28505624

[11] Xu JX Yu JN Wang XJ. Aradiologic diagnostic scoring model based on CT features for differentiating gastric schwannoma from gastric gastrointestinal stromal tumors. Am J Cancer Res. (2022) 12:303–14. PMC8822295 35141019

[12] Zhang Y Mao XL Zhou XB. Long-term outcomes of endoscopic resection for small(≤4.0cm)gastric gastrointestinal stromal tumors originating from the muscularis propria layer. World J Gastroenterol. (2018) 24:3030–7. doi: 10.3748/wjg.v24.i27.3030 PMC6054947 30038470

[13] von Mehren M Joensuu H. Gastrointestinal stromal tumors. J Clin Oncol. (2018) 36:136–143. doi: 10.1200/JCO.2017.74.9705 29220298 PMC6553810

[14] Wang W Cao K Han Y. Computed tomographic characteristics of gastric schwannoma. J IntMedRes. (2019) 47:1975–1986. doi: 10.1177/0300060519833539 PMC6567782 30871392

[15] Annunziata S Treglia G Caldarella C. The role of 18F-FDG-PET and PET/CT in patients with colorectal liver metastases undergoing selective internal radiation therapy with yttrium-90: A first evidence-based review. Sci World J. (2014) 2014:879469. doi: 10.1155/2014/879469 PMC3929576 24672385

[16] Fiorentino A Laudicella R Ciurlia E. Positron emission tomography with computed tomography imaging (PET/CT) for the radiotherapy planning definition of the biological target volume: PART 2. Crit Rev Oncology/Hematology. (2019) 139:117–24. doi: 10.1016/j.critrevonc.2019.03.008 30940428

[17] Gillies JR Kinahan EP Hedvig H. Radiomics: images are more than pictures, they are data. Radiology. (2016) 278:563–77. doi: 10.1148/radiol.2015151169 PMC4734157 26579733

[18] Federica V Roberto C Albert C. Radiomics and artificial intelligence: new frontiers in medicine. Recenti progressi medicina. (2020) 111:130–5. 10.1701/3315.32853 32157259

[19] Levy DA Remotti EH Thompson MW. Gastrointestinal stromal tumors: radiologic features with pathologic correlation. Radiographics: Rev Publ Radiological Soc North America Inc. (2003) 23:283–304, 456; quiz 532. 10.1148/rg.232025146 12640147

[20] Hui Z Haoyan C Shengjian Z. Differentiation of gastric true leiomyoma from gastric stromal tumor based on biphasic contrast-enhanced computed tomographic findings. J Comput assisted tomography. (2014) 38:228–34. doi: 10.1097/RCT.0b013e3182ab0934 24625607

